# Nano-based formulations of thymoquinone are new approaches for psoriasis treatment: a literature review

**DOI:** 10.3389/fimmu.2024.1416842

**Published:** 2024-08-12

**Authors:** Amir Modarresi Chahardehi, Hamid Reza Ojaghi, Hossein Motedayyen, Reza Arefnezhad

**Affiliations:** ^1^ Kimia Andisheh Teb Medical and Molecular Laboratory Research Co., Tehran, Iran; ^2^ Department of Dermatology, Tabriz University of Medical Sciences, Tabriz, Iran; ^3^ Autoimmune Diseases Research Center, Kashan University of Medical Sciences, Kashan, Iran; ^4^ Coenzyme R Research Institute, Tehran, Iran; ^5^ Student Research Committee, Shiraz University of Medical Sciences, Shiraz, Iran

**Keywords:** psoriasis, thymoquinone, topical therapy, nano-thymoquinone, nano-formulation

## Abstract

Psoriasis, a persistent immune-mediated inflammatory skin condition, affects approximately 2-3% of the global population. Current treatments for psoriasis are fraught with limitations, including adverse effects, high costs, and diminishing efficacy over time. Thymoquinone (TQ), derived from *Nigella sativa* seeds, exhibits promising anti-inflammatory, antioxidant, and immunomodulatory properties that could prove beneficial in managing psoriasis. However, TQ’s hydrophobic nature and poor bioavailability have hindered its usefulness as a therapeutic agent. Recent research has strategically addressed these challenges by developing nano-thymoquinone (nano-TQ) formulations to enhance delivery and efficacy in treating psoriasis. Preclinical studies employing mouse models have demonstrated that nano-TQ effectively mitigates inflammation, erythema, scaling, epidermal thickness, and cytokine levels in psoriatic lesions. Various nano-TQ formulations, including nanoemulsions, lipid vesicles, nanostructured lipid carriers, and ethosomes, have been explored to improve solubility, facilitate skin penetration, ensure sustained release, and achieve site-specific targeting. Although clinical trials are currently scarce, the outcomes from *in vitro* and animal models are promising. The potential co-delivery of nano-TQ with other anti-psoriatic agents also presents avenues for further investigation.

## 1 Introduction

Psoriasis, a chronic inflammatory skin disease, affects an estimated 125 million individuals globally, with prevalence rates varying among ethnic groups, typically ranging from 0.33% to 0.6% (1). This autoimmune condition significantly impacts overall health beyond its dermatological manifestations (2). Despite its prevalence, no diagnostic tests are currently available for identifying psoriasis due to its autoimmune nature (3). Characterized by immune-mediated inflammation, psoriasis often persists throughout an individual’s lifetime and is associated with various severe complications (4). It predominantly occurs in middle-aged and elderly individuals, usually during the third to fourth decade of life, with a slight male preference (5). While affecting both genders, it frequently manifests initially in women and individuals with a genetic predisposition (4). Additionally, its incidence is higher in regions with higher income levels and aging populations (6). Psoriasis carries a substantial risk of progressing to psoriatic arthritis, observed in a considerable percentage of patients with plaque-type psoriasis, typically ranging from 7% to 48% (5). The diverse clinical presentations of psoriasis have led to its categorization into multiple subtypes, reflecting the wide range of morphological variations associated with the condition. These subtypes include plaque psoriasis, guttate psoriasis, flexural (inverse) psoriasis, erythroderma, generalized pustular psoriasis, palmoplantar pustulosis, and psoriatic nail disease (7). Psoriasis research has yielded significant advancements in therapeutic strategies. Current treatments aim to control symptoms, improve quality of life, and suppress disease activity (8). Recent successes involve biological therapies targeting interleukin (IL)-17 and IL-23p19, such as Guselkumab, Tildrakizumab, and the IL-12/IL-23 inhibitor Ustekinumab, have demonstrated remarkable efficacy, particularly in managing PsA (7, 9). However, it is crucial to acknowledge the limitations of current therapies. Importantly, they do not offer a cure, and a concerning trend is the increasing number of patients with severe psoriasis who exhibit inadequate responses to available options (9). Additionally, topical therapies face the challenge of poor penetration through the thickened, scaly psoriatic skin (10). This underscores the urgent need for novel therapeutic strategies with minimal therapeutic and pharmacological barriers.

Thymoquinone (TQ), a bioactive compound of *Nigella sativa* seeds, has emerged as a therapeutic candidate for various diseases due to its diverse pharmacological properties (11), including antioxidant, anti-inflammatory, antiviral, anticancer (12, 13), antibacterial (14, 15), immunomodulatory (16), anticoagulant (17), antipsychotic, anxiolytic, antidepressant (18), and anticonvulsant activities (19, 20). Notably, TQ demonstrates promising anti-psoriatic effects (7). However, its hydrophobicity, low aqueous solubility, and photosensitivity limit its clinical application (7). To address these limitations and enhance TQ’s therapeutic potential for psoriasis, researchers are exploring alternative delivery methods. Recent advancements in nanomedicine, particularly nanocarrier-based encapsulation of TQ, offer a promising solution. This approach aims to overcome the bioavailability challenges associated with TQ and unlock its full therapeutic potential in managing psoriasis (4, 7).

Therefore, in light of the limitations associated with current psoriasis therapies and the promising preclinical data on TQ’s anti-psoriatic effects, this review comprehensively evaluates the existing scientific knowledge regarding the therapeutic potential of TQ for psoriasis. We focus on the mechanisms underlying its efficacy and explore the development of nanocarrier-based formulations to overcome TQ’s limitations and enhance its therapeutic application in psoriasis management.

## 2 Potential uses of nanotechnology in dermatology

Nanotechnology has revolutionized drug delivery, particularly in dermatology (21), offering innovative tools for treating skin disorders (22, 23). Amongst various administration routes, topical delivery holds promise for lipophilic drugs like TQ, enabling efficient penetration through the stratum corneum with minimal systemic exposure (24, 25). Nanotechnology further enhances topical delivery by employing diverse nanocarriers, including liposomes, polymeric nanoparticles, and gold nanoparticles. These nanocarriers exploit their size and surface properties to facilitate drug transport across the skin barrier, reaching deeper epidermal and dermal layers (24). Traditionally, the skin’s barrier function poses a significant challenge for drug delivery. However, nanotechnology-based solutions have demonstrated remarkable potential in overcoming this hurdle (21, 26, 27). Cutaneous delivery utilizing nanocarriers allows for targeted drug delivery to specific psoriatic lesions while minimizing systemic side effects, offering substantial benefits in managing inflammatory skin conditions, including acne, inflammation, infections, and wound healing (24, 27). Additionally, the unique characteristics of these nanocarriers, such as their small size (10-1000 nm), surface properties (charge, hydrophobicity), and ability to incorporate targeting ligands, facilitate their penetration through the stratum corneum, the outermost layer of the skin. These features allow them to: (i) passively diffuse between corneocytes (skin cells) due to their small size, (ii) enhance permeation through controlled modifications of surface properties, and (iii) target specific receptors on skin cells using ligands, ultimately leading to deeper delivery of the encapsulated drug. This potential for customization based on disease severity, skin characteristics, and potential allergies paves the way for a more personalized approach to treating psoriasis (28) with nano-thymoquinone (nano-TQ).

**Figure 1:**
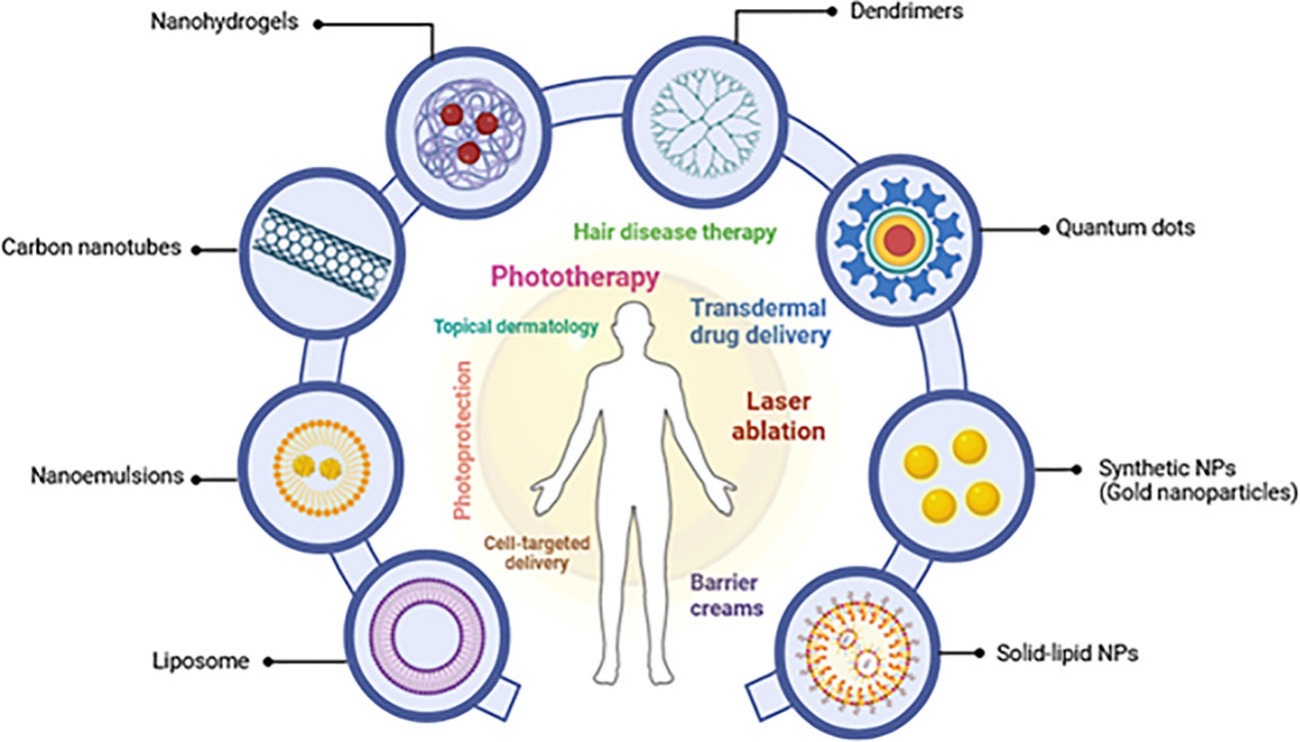
A schematic depicting several nanoparticles and their dermatological topical therapeutic uses (adapted from Souto et al. (2020) and Raszewska-Famielec and Flieger, 2022) (28, 30).

Nanocarriers, including polymeric nanoparticles and lipid-based carriers, have shown a potential to enhance the transportation of medicinal substances to the skin, improving absorption and efficacy in medication delivery for various skin disorders, including alopecia, vitiligo, and psoriasis (28). For instance, lipid nanoparticles (LNs) have emerged as a promising drug delivery system (DDS) for various skin problems due to their biocompatibility and versatility (29). These characteristics of lipids contribute to their therapeutic potential in managing cutaneous diseases (25). Notably, nanostructured lipid carriers (NLCs) have shown promise in delivering medications to psoriatic lesions, potentially improving treatment outcomes for dermatitis, bacterial infections, and even skin cancer (25). Beyond therapeutics, nanotechnology is impacting dermo-cosmetics. Liposome-containing moisturizers were among the first cosmetic products to utilize this technology (30). Additionally, advancements in non-invasive nanoimaging techniques using gold nanoparticles, quantum dots, and magnetic nanoparticles are revolutionizing diagnostic modalities in dermatology (23), as illustrated in **Figure 1**. Despite its immense potential, unresolved issues concerning nanotechnology in dermatology remain. These include a thorough investigation of potential health risks associated with nanoparticles and a deeper understanding of their skin penetration mechanisms (28).

## 3 Understanding psoriasis

The epidermis is susceptible to various pathological changes, encompassing inflammatory, neoplastic, traumatic, hormonal, degenerative, and even inherited conditions (29). As mentioned previously, psoriasis, a prevalent and chronic skin disorder characterized by inflammation, is strongly associated with oxidative stress, abnormal epidermal proliferation, infiltrating inflammatory cells, and increased angiogenesis in the dermis. Crucial treatments for psoriasis focus on antioxidation and suppressing aberrant keratinocyte growth (31). This chronic inflammatory skin disorder manifests as painful red or white itchy scales or plaques (32), with its incidence reaching 11% among Caucasian and Scandinavian populations (33) while being less common in Asians and Africans (33).

Psoriasis pathophysiology has been extensively studied, particularly following the establishment of the European Society for Dermatological Research. Research has identified the crucial role of various skin layers and the interplay between innate and acquired immunity in disease development, often depicted through a tree-like model (34). The overall severity of psoriasis is influenced by various factors, including disease duration, lesion location, inflammation levels, treatment response, and quality of life impact. Importantly, classification of psoriasis severity often relies on inclusion criteria used in randomized controlled clinical trials. Common metrics for assessing chronic plaque psoriasis severity include the Psoriasis Area and Severity Index (PASI), Body Surface Area (BSA), and Physician Global Assessment (PGA) (35).

**Figure 2:**
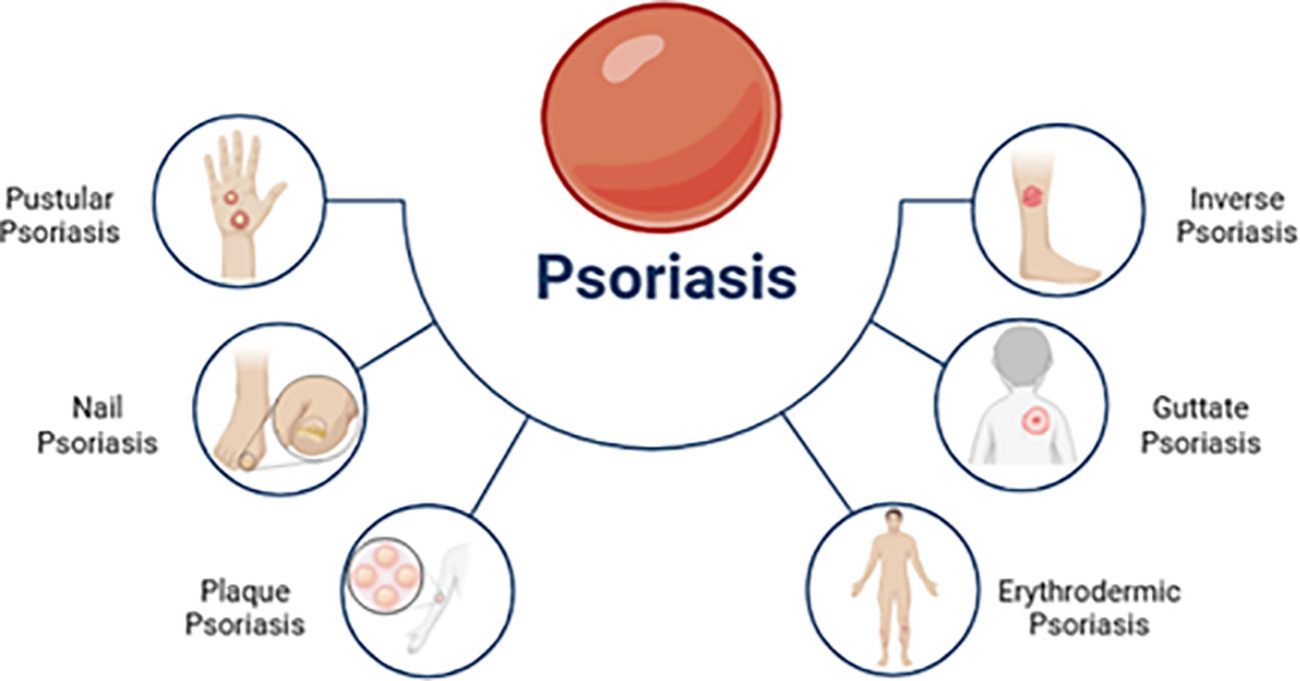
Several clinical subtypes of psoriasis [adapted from Ahmad et al. (2023)] (36).

Psoriasis presents with diverse clinical manifestations. Plaque psoriasis, or psoriasis vulgaris, is the most common form. However, other subtypes exist, each with distinct characteristics relevant to therapeutic approaches. **Figure 2** illustrates this heterogeneity. Understanding these subtypes is crucial for optimizing treatment strategies, including (33, 37, 38):

1. Psoriasis vulgaris: This chronic plaque-type accounts for 90% of psoriasis cases. It is characterized by sharp, erythematous, and pruritic plaques covered in silvery scales commonly found on the trunk, limbs, and scalp.

2. Guttate psoriasis: Guttate psoriasis is marked by the sudden appearance of small erythematous plaques. Group-A streptococcal tonsil infections often trigger it, particularly in children and teenagers.

3. Inverse psoriasis: Also known as flexural psoriasis, this subtype causes slightly erosive erythematous plaques and patches in intertriginous areas.

4. Pustular psoriasis: Pustular psoriasis is characterized by the formation of multiple sterile pustules. It can manifest as localized or widespread pustules. Localized phenotypes include PPP (palmoplantar pustulosis) affecting the palms and soles and acrodermatitis continua Hallopeau (ACS) affecting the nail apparatus at the points of fingers and toes.

5. Erythrodermic psoriasis: A very uncommon and severe form of psoriasis vulgaris, erythrodermic psoriasis (EP) affects an estimated 1% to 2.25 percent of psoriatic individuals. Histopathologic and clinical features of the disorder are different, and one of them is a widespread inflammatory erythema that covers at least 75% of the body’s surface.

6. Nail psoriasis: Nail psoriasis often arises due to psoriatic inflammation that affects either the nail bed or the nail matrix. Mild to severe hyperkeratosis, spongiosis, and localized hyperkeratosis are histopathological findings of nail psoriasis, which are comparable to those of cutaneous psoriasis.

### 3.1 Pathophysiology of psoriasis

Psoriasis pathogenesis involves an intricate interplay of cellular and molecular events, ultimately leading to the development of characteristic psoriatic lesions.

#### 3.1.1 Contributing factors

Psoriasis pathogenesis is a complex interplay of genetic and environmental factors. While the exact mechanisms remain under investigation, several key contributors have been identified. Genetic susceptibility plays a role, with HLA-Cw6 and CARD14 gene mutations increasing the risk. Aside from infectious diseases, medicines, and lifestyle choices, environmental risk factors have also been associated (10, 39), which we will now highlight as significant variables.

#### 3.1.2 Key factors

##### 3.1.2.1 Genetic factors

Multiple genes and loci contribute to the susceptibility and pathogenesis of psoriasis, creating a complex genetic landscape. The first gene strongly associated with psoriasis susceptibility is HLA-Cw6, located at PSORS1 on chromosome 6p21.3 (40). The HLA-Cw6 allele is part of the major histocompatibility complex class I, and proper priming for cell lysis targeted by activated CD8+ T-cells requires MHC class I. This investigation supports the centrality of T cells in the etiology of psoriasis. However, HLA-Cw6 is not the sole molecule involved in antigen presentation linked to psoriasis. A recent genome-wide association study (GWAS) found that individuals with the HLA-Cw6 mutation were more likely to have a role for the ERAP1 gene (41). Psoriasis has been linked to genes that encode components of the IL-23/IL-12 pathways, including IL23R and IL12B. Th17 cells, which play a role in the inflammatory processes seen in psoriasis, rely on these cytokines for their development and maintenance (42). Li et al. found that only one of the seven single nucleotide polymorphisms (SNPs) in the six IL genes—rs3212227 in the IL12B gene—was determined to be genotypically linked with psoriasis (42). On the other hand, tumor necrosis factor alpha-induced protein 3 (TNFAIP3) and TNFAIP3 interacting protein 1 (TNIP1) are genes that play a role in the nuclear factor-kappa B (NF-κB) signaling system, which is an important regulator of inflammation and immunological responses. Psoriasis has been linked to variations in these genes, emphasizing the role of NF-κB signaling in the condition (43). For instance, According to Indu’s findings, the TNIP1 gene (SNP rs17728338) and the TNFAIP3 gene (SNP rs610604) were both genotypically and allelically linked to psoriasis in the South Tamil Indian population (43). A gene called endoplasmic reticulum aminopeptidase 1 (ERAP1) has been associated with psoriasis. This gene plays a role in peptide trimming for MHC class I presentation, and variations in ERAP1 may affect the immune response to self or foreign antigens, which might be associated with the pathogenesis of psoriasis (44).

##### 3.1.2.2 Immune factors

Interactions between tumor necrosis factor-alpha (TNF-α), interferon-gamma (IFN-γ), and IL-17 induce inflammation and epidermal hyperproliferation (45). Elevated levels of inflammatory cytokines such as TNF-α and IL-6 have been linked to reduced sleep in psoriasis (46). Psoriatic plaques exhibit acanthosis (epidermal hyperplasia) and inflammatory infiltrates composed of dermal dendritic cells, macrophages, T cells, and neutrophils histologically (33). Increased levels of inflammatory cytokines such as TNF-α and IL-6 are associated with reduced sleep duration in psoriasis patients. Additionally, disrupted sleep cycles and regulation have been linked to higher substance P concentrations in the perilesional skin of psoriatic patients (46). Several pieces of evidence have led scientists to conclude that T cells play a significant role in the development of psoriasis. Initial psoriatic lesions exhibit a high concentration of T cells (34). The etiology may involve abnormal expressions of E3 ubiquitin ligase and a dysregulated protein ubiquitination pathway (47).

##### 3.1.2.3 Environmental factors

Beyond genetic predisposition, environmental factors significantly impact the onset, severity, and course of psoriasis (39, 48) as following:

1. Ultraviolet (UV) Light: Exposure to UVB radiation, a component of sunlight, is a well-established environmental trigger. UVB exposure can induce inflammatory mediators, activate the immune system, and ultimately lead to psoriatic lesion formation (49).
2. Smoking: Cigarette smoking is another significant environmental risk factor associated with psoriasis. The presence of various toxins and inflammatory compounds in cigarette smoke contributes to the underlying inflammatory processes in psoriasis (50).
3. Stress: Numerous studies highlight the link between stress and psoriasis flares. Stress can exacerbate existing psoriasis or even trigger its onset (51).
4. Obesity: Adipose tissue in obese individuals can promote the production of pro-inflammatory cytokines, potentially contributing to the chronic inflammation characteristic of psoriasis (52).
5. Infections: Some researchers suggest a possible role for infections, particularly those involving streptococcal bacteria, in triggering guttate psoriasis (53).
6. Other environmental factors: While the evidence is less conclusive, alcohol consumption and certain medications have also been implicated in psoriasis pathogenesis (53).

### 3.2 Current psoriasis treatment landscape: progress and challenges

Psoriasis treatment strategies are tailored to disease severity, patient characteristics, and treatment efficacy and safety profiles (35). Recent advancements include combination therapy, novel biologics targeting new pathways, and gene/cell-based therapies (8). While topical therapies are preferred for mild cases, biologics have revolutionized treatment for moderate to severe psoriasis by targeting specific disease mechanisms (8). Biologics, despite their effectiveness, are often reserved as a last resort due to limitations like cost and potential side effects (54, 55). Hence, significant progress has been made in psoriasis treatment with the introduction of several therapeutic approaches, such as:

#### 3.2.1 Conventional systemic treatments

Psoriasis is traditionally treated with methotrexate, a systemic medicine known for its cytotoxic, anti-inflammatory, and immune-modulatory effects, making it therapeutically valuable. However, methotrexate is associated with potentially hazardous consequences and various issues. For decreasing side effects, new colloidal drug delivery techniques for methotrexate have been developed. These systems include hydrogel, nanoparticles, niosomal gel, liposomal formulation, albumin conjugates, and NLCs. These novel delivery methods aim to reduce the drug’s harmful effects in psoriatic patients by enhancing skin permeability while decreasing systemic availability (56).

#### 3.2.2 Topical treatments

The recent licensing of Tapinarof, a topical agent targeting the aryl hydrocarbon receptor (AhR), represents a significant advancement in psoriasis treatment. AhR modulates skin barrier function and inflammatory responses, making it a promising therapeutic target for psoriasis. Tapinarof demonstrates superior efficacy and a favorable safety profile compared to some existing topical medications. This is particularly noteworthy as current topical treatments for psoriasis may be limited by safety concerns, high costs, or frequent dosing requirements. Tapinarof offers a promising alternative with enhanced efficacy and improved tolerability for psoriasis management (57).

#### 3.2.3 Biological medical therapies

Psoriasis pathogenesis involves dysregulation of the immune system, leading to the production of inflammatory cells and cytokines. Biological agents represent a significant advancement in psoriasis treatment by specifically targeting these immune pathways. These therapies effectively manage psoriatic inflammation and symptoms. However, a crucial consideration is the potential for immunosuppression, which necessitates careful monitoring. **Table 1** summarizes the various biological agents used in psoriasis treatment.

**Table 1:** Biological approaches for psoriasis treatment.

Biological therapies	Examples	Mechanisms of action	Ref.
TNF-α inhibitors	Adalimumab (Humira), Etanercept (Enbrel), Infliximab (Remicade), and Certolizumab pegol (Cimzia)	TNF-α is essential for inflammation and immune cell activation. Blocking TNF-alpha reduces inflammation and psoriatic plaque development	(58)
IL-17 inhibitors	Secukinumab (Cosentyx) and Ixekizumab (Taltz)	IL-17 induces another inflammatory cytokine and chemokine. Delaying IL-17 activation reduces inflammation and psoriatic plaque development	(59)
IL-12/23 inhibitors	Ustekinumab (Stelara)	Cytokines, such as IL-12 and IL-23, which are involved in psoriasis, encourage the development and function of T cells. Reduced T cell activation and psoriatic plaque development can be achieved by blocking the activities of IL-12 and IL-23 with IL-12/23 inhibitors.	(60, 61)
Anti-IL-23 inhibitors	Guselkumab (Tremfya)	The cytokine IL-23 is essential for the development of psoriasis because it stimulates the differentiation and activation of a subgroup of T cells called Th17 cells. Inhibitors of IL-23 can decrease the activation of Th17 cells and the creation of psoriatic plaques by blocking the activity of IL-23.	(62)
T Cell Costimulation Blockers	Abatacept (Orencia)	Psoriatic plaque development and Th17 cell activation are both reduced with the use of IL-23 inhibitors, which work by blocking the action of IL-23.	(63)
Integrin Inhibitors	Apremilast (Otezla)	Immune cells bind to other cells and tissues using integrins. Integrin inhibitors stop immune cells from entering the skin and lowering psoriatic plaque development.	(64)
Phosphodiesterase 4 (PDE4) Inhibitors	Apremilast (Otezla)	The enzyme PDE4 degrades cyclic AMP (cAMP), which regulates immune cell activity. PDE4 inhibitors increase cAMP, suppressing immune cell activity and psoriatic plaque formation.	(64)
AK Inhibitors	Tofacitinib (Xeljanz) and Baricitinib (Olumiant)	JAK enzymes carry cell surface receptor signals within cells. JAK inhibitions alter inflammatory signaling pathways and reduce psoriatic plaque formation.	(65, 66)

#### 3.2.4 Adjuvant therapies

Metformin, a first-line antidiabetic drug, is being explored as an adjuvant treatment for generalized psoriasis alongside methotrexate. Its anti-inflammatory and antiproliferative properties, similar to those of methotrexate, suggest potential benefits in improving metabolic syndrome characteristics in psoriatic patients (67). This combination therapy may lead to enhanced clinical outcomes. It is important to note that the initial management of moderate-to-severe psoriasis often relies on traditional systemic medications, including methotrexate, cyclosporine, acitretin, and phototherapy with UVB 311nm or PUVA radiation (68).

However, several challenges remain:

- Patient adherence: Many patients struggle with adherence due to concerns about treatment efficacy (56).
- Limited effectiveness for moderate/severe cases: More effective interventions are needed for moderate to severe psoriasis (57).
- High cost of biologics treatment: The high cost of biologics and the need for long-term treatment limit accessibility
- Topical treatment limitations: Current topical treatments can have poor safety profiles, high costs, and frequent dosing requirements (57).

On the other hand, researchers are exploring new avenues to address these challenges. Examples include:

- Novel delivery methods for existing drugs: Enhancing the efficacy and safety of existing drugs like methotrexate through improved delivery methods (68).
- Next-generation topical medications: New topical medications like Tapinarof offer improved efficacy and safety profiles compared to current options (57).
- Combination therapies: Combining existing drugs to improve efficacy and reduce side effects (8).
- Biologics targeting new pathways: Developing biologics that target novel pathways involved in psoriasis pathogenesis (8).
- Gene and cell-based therapies: Investigating the potential of gene and cell-based therapies for long-term disease control (8).

Addressing current limitations and actively investigating novel treatment avenues are crucial endeavors in psoriasis research. These efforts ultimately aim to improve the quality of life for patients living with this chronic condition.

### 3.3 Nanocarriers and targeted nanoparticles in psoriasis treatment

Nanotechnology has emerged as a promising avenue in dermatology, offering common strategies for treating psoriasis, a chronic inflammatory skin condition (69). Compared to traditional medications, nanocarriers and targeted nanoparticles provide several advantages, including enhanced drug penetration into the skin, controlled drug release, and potentially reduced systemic side effects.

#### 3.3.1 Nanocarriers for topical psoriasis treatment

The topical administration of antipsoriatic drugs using nanocarriers, such as liposomes, niosomes, and polymeric nanoparticles, has garnered significant research interest. These nanocarriers have the ability to target the damaged skin regions with delivery, increase skin penetration, and improve medication solubility.

1. Liposomes: Liposomes are nanocarriers made of lipids that have the ability to encapsulate medicines that are hydrophilic or hydrophobic. In both animal models and human clinical studies, methotrexate and cyclosporine A liposomal formulations significantly reduced psoriatic lesions (70). However, lipid-based nanocarriers, including liposomes and solid lipid nanoparticles (SLNs), have demonstrated encouraging outcomes in the treatment of psoriasis. These systems have the potential to efficiently contain both water-soluble and fat-soluble medications, enhancing their durability and ability to penetrate the skin. For instance, a study by Pradhan et al. showed that SLNs containing methotrexate were more effective in decreasing psoriatic plaques compared to traditional methotrexate formulations (71).
2. Niosomes: Niosomes are vesicles composed of non-ionic surfactants that provide benefits compared to liposomes, including enhanced stability and decreased expense. Preclinical investigations have shown that niosomal formulations of tazarotene and calcipotriol have improved skin penetration and anti-inflammatory properties (72).
3. Polymeric Nanoparticles: Polymeric nanoparticles have garnered interest because of their ability to be compatible with living organisms and their wide range of applications. Poly(lactic-co-glycolic acid) (PLGA) nanoparticles containing tacrolimus have demonstrated increased epidermal penetration and extended drug release, potentially enhancing the therapeutic effectiveness of the medicine in treating psoriasis (73). Polymeric nanoparticles, composed of polymers that may degrade naturally and are compatible with living organisms, offer the ability to release drugs in a controlled manner and deliver them to specific targets. PLGA nanoparticles containing vitamin D3 analogues have demonstrated enhanced treatment effectiveness and decreased systemic absorption in animal models of psoriasis (74).

Recent progress in targeted nanocarriers has enhanced the precision of psoriasis therapies. Nanoparticles functionalized with peptides targeting receptors overexpressed in psoriatic skin have shown increased drug accumulation in the afflicted regions. A study conducted by Deng et al. found that nanoparticles loaded with cyclosporine A and coupled with a cell-penetrating peptide had markedly enhanced anti-psoriatic effects in comparison to nanoparticles that were not specifically targeted (75). Furthermore, the use of stimuli-responsive nanocarriers has surfaced as a highly promising method. These intelligent systems have the ability to release their payload when certain triggers are detected in the psoriatic microenvironment, such as an increase in pH or the presence of excessively produced enzymes. Doppalapudi et al. created chitosan nanoparticles that respond to changes in pH and are capable of delivering clobetasol propionate. These nanoparticles were shown to release the medicine more effectively under the alkaline conditions commonly found in psoriatic lesions (76).

#### 3.3.2 Targeted nanoparticles for psoriasis treatment

Targeted nanoparticles are engineered to precisely transport medications to particular cells or tissues, reducing unintended side effects and improving the effectiveness of treatment. Targeted nanoparticles can be designed to specifically target activated T cells or inflammatory cytokines in psoriasis (77).

1. Antibody-conjugated nanoparticles: Nanoparticles conjugated with antibodies that specifically recognize activated T cell markers, such as CD4 and CD8, have the ability to specifically target and transport medicines to disease-causing T cells in psoriatic lesions. This technique has demonstrated encouraging outcomes in preclinical investigations, diminishing inflammation and enhancing skin histology (78).
2. Cytokine-targeted nanoparticles: Nanoparticles can be engineered to specifically target and counteract crucial inflammatory cytokines implicated in the development of psoriasis, such as TNF-α and IL-17. Nanoparticles that are bound to anti-TNF-α antibodies or contain IL-17 inhibitors have shown improved effectiveness in treating psoriasis in animal models (79).

## 4 Thymoquinone: properties and effects in medical approaches

**Figure 3:**
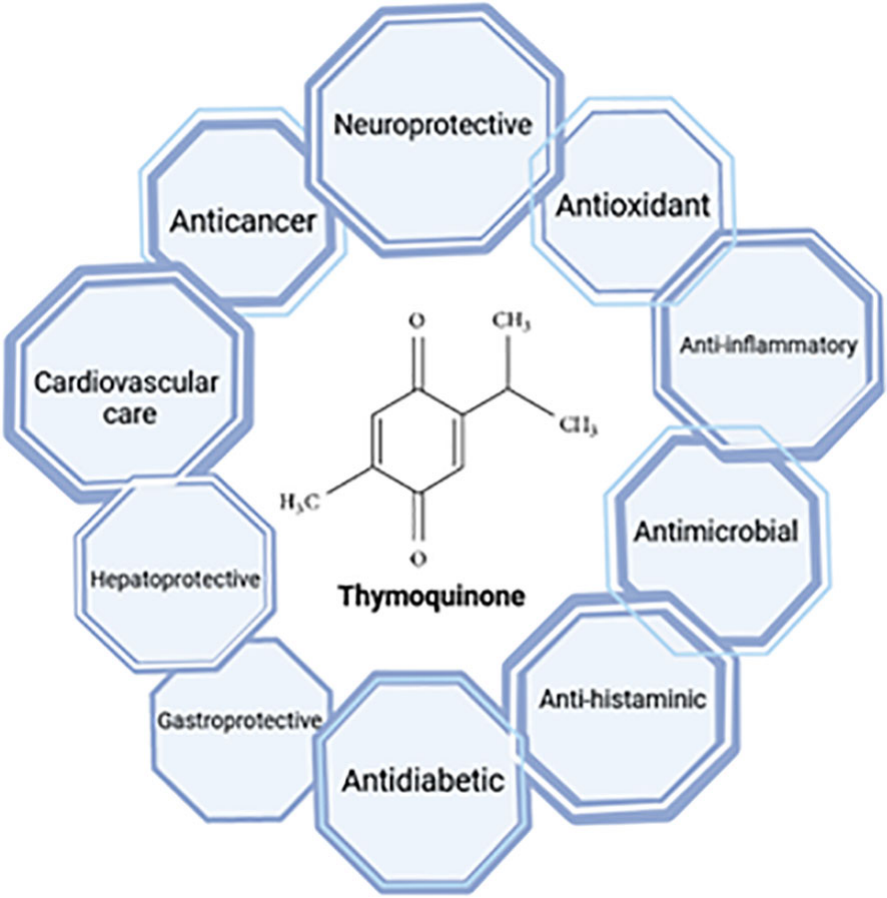
Thymoquinone and its medical approaches.

TQ (2-isopropyl-5-methylbenzo-1, 4-quinone, Molecular Weight: 164.2) is primarily extracted from the triangular seeds of *N. sativa* and their essential oils (80). Initially discovered in 1960, TQ was identified as a yellow precipitate with slight solubility in water but miscibility with ethanol, dimethyl sulfoxide, and dimethyl formamide. Emerging research highlights the multifaceted therapeutic potential of TQ, encompassing anti-inflammatory, anti-tumor, and free radical scavenging activities. Additionally, TQ has been shown to modulate the immune system and protect vital organs, including the liver and heart, as illustrated in **Figure 3** (80).

### 4.1 Thymoquinone features

#### 4.1.1 Anti-inflammatory and immunomodulatory properties

TQ exerts its anti-inflammatory effects through a complex interplay of signaling pathways and molecular mechanisms, including antioxidant activity that mitigates oxidative stress (80). Extensive research has revealed that TQ has anti-inflammatory effects across various conditions, ranging from sepsis and rheumatoid arthritis to obesity and viral infections (81–83). Notably, TQ’s anti-inflammatory effects involve suppressing key pro-inflammatory mediators, including IL-2, IL-4, IL-6, and IL-12, while simultaneously promoting the production of IFN-γ, a cytokine with immunomodulatory functions (16).

Beyond its ability to suppress pro-inflammatory cytokines, TQ exhibits additional immunomodulatory properties relevant to skin conditions. Studies have demonstrated its anti-inflammatory effects through the inhibition of NF-κB activation and cyclooxygenase-2 (COX-2) production. Additionally, TQ stimulates the production of human beta-defensin-3 (hBD-3), antimicrobial peptides (AMPs), and LL-37, which contribute to skin barrier integrity and defense against pathogens. Furthermore, TQ influences the production of free fatty acids, ceramides, and other immune-related factors in the skin (80). These findings, particularly the modulation of hBD-3 and AMPs, suggest TQ’s potential to regulate the immune response in atopic dermatitis (AtD), a chronic inflammatory skin condition (84).

**Figure 4:**
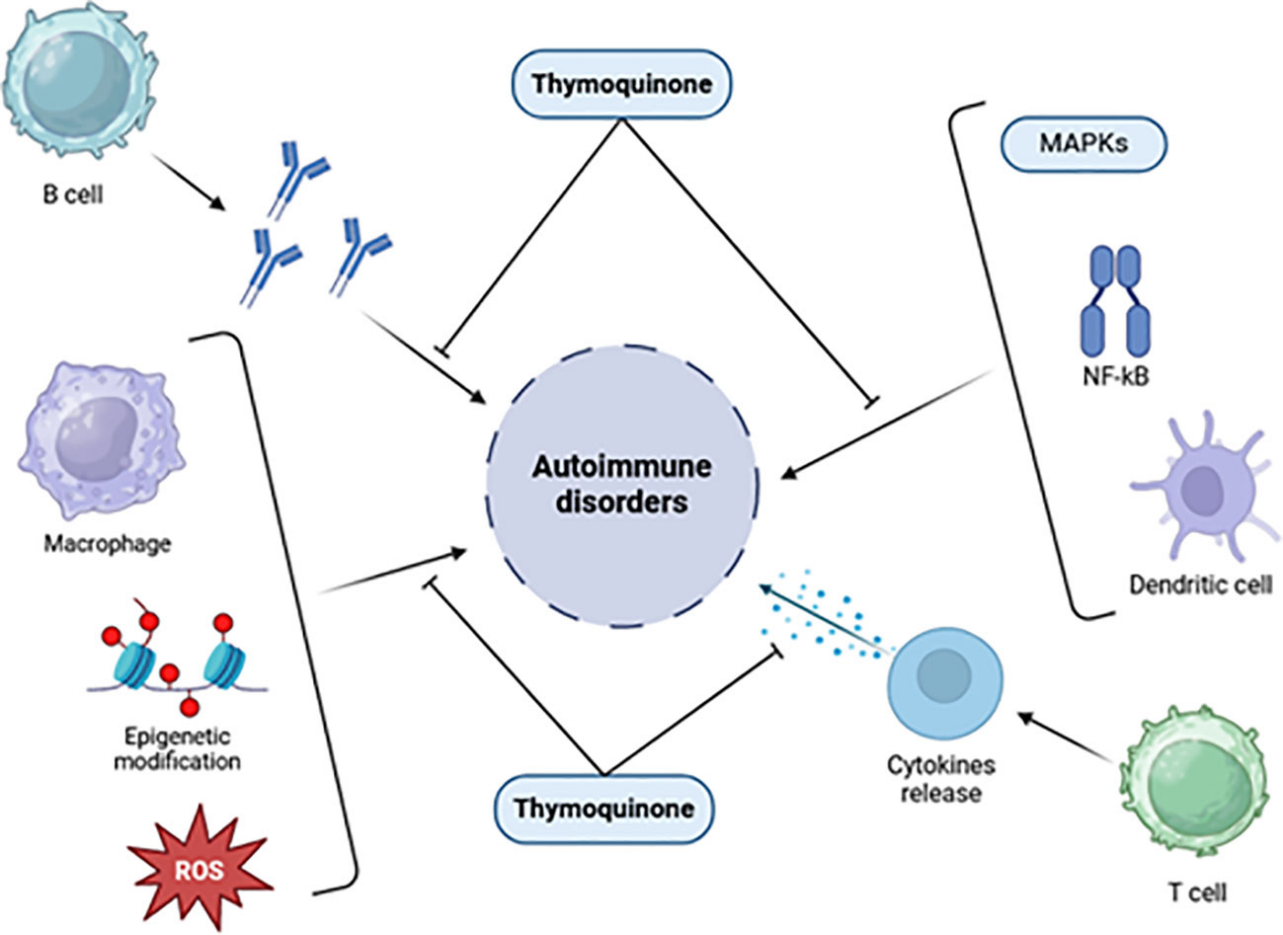
Effects of thymoquinone on inflammatory chemicals, immunological cells, signaling pathways, and epigenetic machinery [adapted from Ali et al. (2021)] (85).

**Figure 4** highlights the potential of TQ to regulate molecules involved in inflammation at the molecular level. TQ exhibits the ability to modulate interferons, interleukins, TNF-α, oxidative stress, regulatory T cells, and various signaling pathways such as NF-κB, Janus kinase/signal transduction and activator of transcription (JAK-STAT), and mitogen-activated protein kinase (MAPK). Given the critical involvement of these molecules and signaling pathways in impaired immunological function, the potential of TQ to regulate these elements holds significance for treating autoimmune disorders. By regulating these molecules and restoring disrupted pathways, TQ may play a valuable role in modulating immune responses and inflammatory reactions associated with autoimmune dysfunction (80, 84).

Due to its anti-inflammatory effects, TQ inhibits the production of key mediators involved in asthma and inflammatory processes, such as 5-lipoxygenase, COX, prostaglandin D2, and leukotrienes. TQ reduces proinflammatory cytokines like interleukins and TNF-α, similar to the effects observed with lipopolysaccharide (LPS). Furthermore, TQ exhibits immunomodulatory properties that influence both cellular and humoral immunity (16). These combined effects suggest the therapeutic potential of TQ in various immune-related disorders, including asthma and autoimmune diseases (16).

#### 4.1.2 Antioxidant properties

While exhibiting both antioxidant and pro-oxidant activities, TQ’s well-documented antioxidant properties make it a promising candidate for mitigating cellular damage and oxidative stress in various diseases (86). Its strong antioxidant activity is linked to its potential for reducing lipid peroxidation, a process known to contribute to cellular damage and inflammation (87). Additionally, studies have shown TQ’s ability to upregulate the expression of antioxidant enzymes like glutathione peroxidase, further protecting cells from oxidative stress in disease models (88). The antioxidant action of TQ protects cells through several mechanisms. It boasts potent free radical scavenging capabilities, thereby reducing oxidative stress and safeguarding cells from reactive oxygen species (ROS) (86, 88). Furthermore, TQ enhances the activity of natural antioxidant defense systems, including superoxide dismutase (SOD) and catalase, while also maintaining optimal glutathione levels. These combined activities play a crucial role in mitigating oxidative stress and protecting cellular components from oxidative damage (88). Studies employing electron spin resonance (ESR) spectroscopy have shown that thymohydroquinone has a higher ability to scavenge radicals compared to TQ.

**Figure 5:**
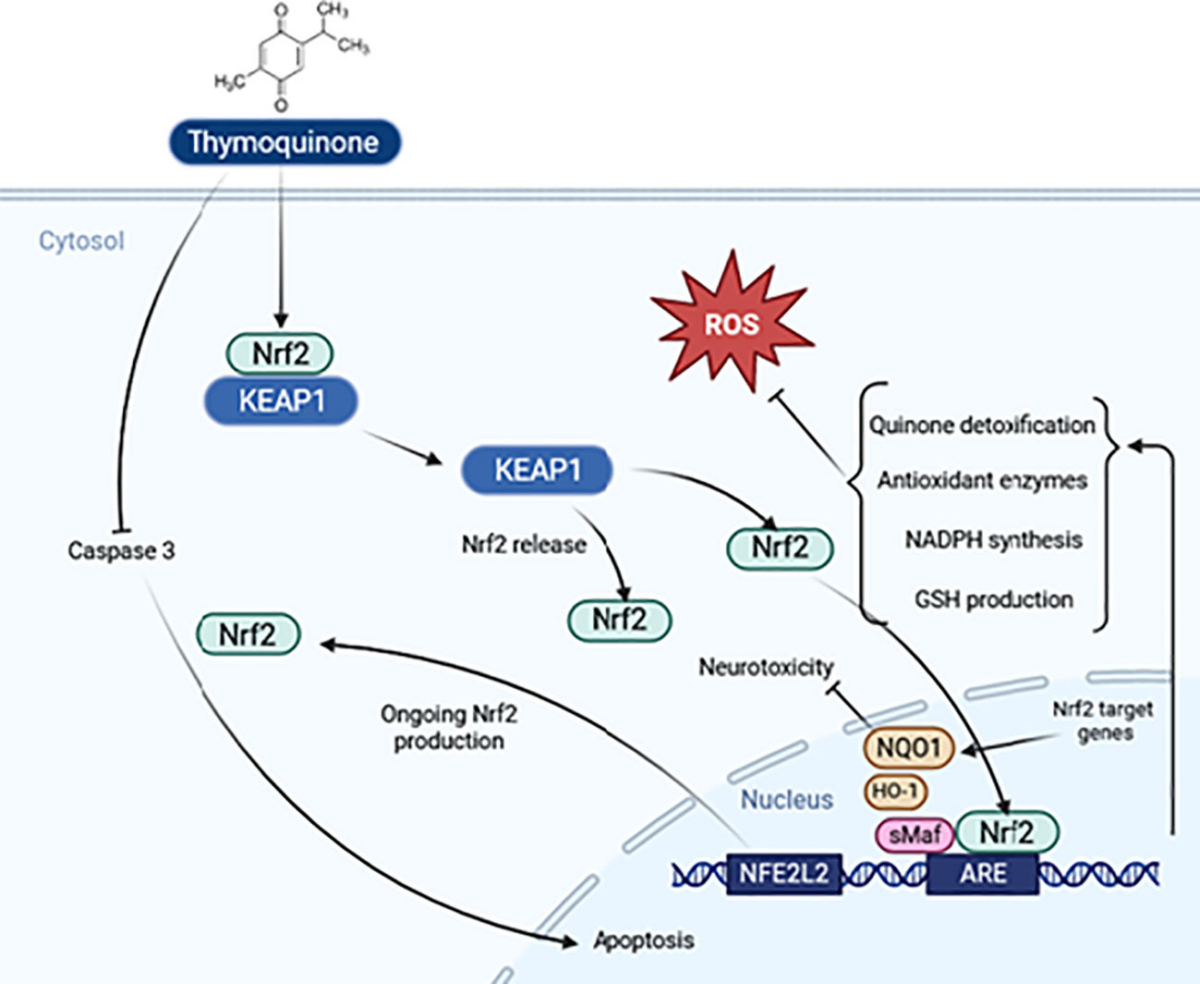
Schematic representation of the thymoquinone pathway and its potential effects on Nrf2 signaling.

The drug’s dosage can be improved by creating mitochondria-targeting antioxidants, using TQ as the antioxidant part. This form of the drug penetrates mitochondria and accumulates under the inner mitochondrial membrane’s electric field. Further research is needed to understand the mechanism of inhibition of oxidative stress by TQ. Also, the nuclear factor-erythroid 2 related factor 2 (Nrf2)- antioxidant responsive element (ARE) axis is a significant antioxidant defense system that has shown neuroprotective benefits (89). The protective effects of TQ under oxidative stress are largely mediated by stimulating the expression of the Nrf2 gene and inducing nuclear translocation of Nrf2, as depicted in **Figure 5**.

#### 4.1.3 Neuroprotective activity

TQ exhibits promising therapeutic potential in various neurological disorders, primarily attributed to its antioxidant and anti-inflammatory properties (18). Mechanistically, TQ modulates endoplasmic reticulum (ER) stress and apoptotic pathways, alleviating excitotoxicity and neuronal damage (90). It also counteracts oxidative stress, downregulates pro-inflammatory cytokine expression, preserves mitochondrial membrane potential, and inhibits caspases-3, -8, and -9, thereby preventing neuronal apoptosis (91). IL-6, IL-12p40/70, and granulocyte colony-stimulating factor (G-CSF) are pro-inflammatory cytokines and chemokines that were discovered to be reduced by TQ. By preventing NF-kB activation increase and its binding to DNA, TQ reduces neuroinflammation. In addition, TQ therapy enhances the binding of Nrf2 to the ARE by inhibiting the inflammation caused by NF-kκB in microglia. TQ acts by interfering with the signaling pathways of phosphoinositide 3-kinase (PI3K)/protein kinase B or NF-kκB, hence inhibiting LPS-induced inflammation of microglia (18). In Alzheimer’s disease (AD), oxidative stress and neuroinflammation are key pathological drivers. TQ’s antioxidant and anti-inflammatory properties position it as a potential therapeutic candidate for AD (88). Alhibshi et al. (2019) demonstrated that TQ protects against amyloid beta-induced neurotoxicity *in vitro*, suggesting its potential application in AD treatment (92). As an antiepileptic, TQ also acts on the central nervous system. It has been observed that TQ protects neurons from Aβ-induced damage in PC12 cells and reduces inflammation associated with stress in AD (18). Furthermore, Abo Mansour et al. (2020) reported that TQ ameliorates cognitive impairment and behavioral deficits in a scopolamine-induced rat model of AD, providing additional evidence for its therapeutic potential (93).

TQ’s therapeutic potential in other neurological disorders is also being explored. For example, studies have shown that TQ can mitigate neuroinflammation and oxidative damage in models of Parkinson’s disease (PD) and Huntington’s disease (HD) (18, 94). Additionally, TQ has been shown to promote neurogenesis and improve cognitive function in animal models of stroke and traumatic brain injury (TBI) (95). One possible explanation for the protective effect of TQ in traumatic brain damage is its ability to decrease oxidative stress in the brain. Nevertheless, a further investigation revealed that while modest doses of TQ demonstrated a neuroprotective impact following severe traumatic brain damage, as evidenced by a reduction in neuronal swelling and partial maintenance of Na+/K+-ATPase function, TQ did not influence the concentrations of glutathione and malondialdehyde (96).

#### 4.1.4 Anticancer activity

Laboratory studies have revealed TQ’s broad-spectrum anticancer activity against various cancer cell lines, including those derived from the larynx, lung, breast, colon, leukemia, ovary, and osteosarcoma (97–99). The anticancer potential of TQ is attributed to its ability to target multiple cellular pathways involved in tumorigenesis. These pathways include malignant development, carcinogenesis, migration, invasion, and angiogenesis (99). Mechanistically, TQ influences various signaling cascades crucial for cancer progression, such as the PI3K/Akt/mTOR pathway, NF-κB pathway, and MAPK pathway (100). In order to meet the anabolic demands of cancer cells as they multiply, several uncontrolled metabolic pathways rely on the PI3K pathway, which is highly active in cancer (101). Additionally, TQ phosphorylated JNK and the extracellular signal-regulated kinase (ERK), causing MAPK to initiate apoptosis. Numerous studies have demonstrated that the MAPK signaling pathway significantly affects TQ antineoplastic properties. The MAPK families include classical MAPK (ERK), C-Jun N-terminal kinase/stress-activated protein kinase (JNK/SAPK), and p38 kinase (102). Studies have demonstrated that TQ promotes apoptosis of cancer cells by regulating the expression of pro-apoptotic and anti-apoptotic genes. Additionally, it reduces the activity of NF-κB and IκB kinase (IKK), which are involved in metastasis, and inhibits ERK1/2 and PI3K pathways (103). El-Baba et al. demonstrated that the tiny molecule TQ binds directly to PAK1, altering its conformation and scaffold function. Future anticancer treatments should investigate combining TQ with targeted therapies due to its effect on the central RAF/MEK/ERK1/2 pathway (104). The activation of AMPK by TQ has been seen to elicit many anti-inflammatory effects, including the upregulation of genes associated with antioxidant properties. Another process involves elevating the concentration of NAD+, which triggers the activation of Sirtuin 1, leading to the deacetylation of p65 and subsequent reduction of NF-κB. Additionally, it will enhance the accumulation of Nrf2, a transcription factor recognized for its antioxidant properties (18). For instance, in triple-negative breast cancer cells, TQ’s antioxidant activity upregulates Nrf2 and downregulates programmed death-ligand 1 (PD-L1), potentially contributing to its therapeutic effects (86). Furthermore, TQ has shown promise in leukemia by reversing DNA hypermethylation in leukemia cells, suggesting its potential role in inhibiting leukemia development (99).

### 4.2 Thymoquinone and treatment of skin diseases

Research is actively exploring the therapeutic potential of TQ in various skin disorders (105). AtD and wound healing are two areas of particular interest where studies have identified potential targets and mechanisms for TQ’s beneficial effects.

#### 4.2.1 Wound healing

The beneficial effects of TQ on skin wound healing include:

- Regulation of inflammation: TQ modulates the inflammatory response, promoting a favorable environment for wound healing.
- Prevention of infection: TQ exhibits antimicrobial and antifungal properties, protecting the wound site from infection.
- Encouragement of tissue regeneration: TQ stimulates the proliferation and migration of cells involved in tissue repair, promoting the formation of new tissue (106, 107).

Kmail et al. showed that TQ can speed up the healing process of wounds through various methods, including influencing the immune system and preventing oxidative damage. TQ may have further therapeutic uses after phase I clinical research confirmed its safety in healthy individuals (106). Moreover, Liang et al. demonstrated that TQ protects human skin keratinocytes from UVA irradiation-induced oxidative stress, inflammation, and mitochondrial dysfunction, suggesting its potential application in preventing and treating UVA-mediated skin damage (108). In a study on wounded animals, a topical nanoemulgel containing TQ showed faster and earlier healing, comparable to a commercially available silver sulfadiazine cream. Histopathological analysis revealed well-organized collagen fibers in the healed tissue, further supporting TQ’s role in promoting tissue repair and suggesting TQ’s potential applications in wound healing (107, 109).

#### 4.2.2 Atopic dermatitis

AtD is a chronic inflammatory skin disease affecting a significant portion of the global population, with estimates suggesting up to 10% of adults and 20% of children experiencing AtD. Clinically, AtD manifests as dryness, erythema, and intense itching. Histologically, it is characterized by spongiosis and infiltration of inflammatory cells in the upper dermis. Studies suggest that TQ holds promise for treating AtD due to its anti-inflammatory and immunomodulatory effects (110). Studies have explored the efficacy of both oral and topical TQ administration in AtD patients. Additionally, animal models have demonstrated a decrease in ear thickness and immunoglobulin E levels with TQ therapy, further supporting its potential to alleviate AtD symptoms (84). While the available data is promising, further investigation is necessary to comprehensively understand the long-term effects of TQ use in treating skin diseases, including AtD (85, 106). These findings suggest potential applications of TQ in enhancing the treatment of AtD and accelerating wound healing (84, 107, 109). Recognizing the potential negative consequences linked to the extended utilization of any medicinal substance is of utmost importance.

### 4.3 Anti-psoriatic properties of TQ

As mentioned previously, TQ has features that render it a promising option for the therapy of autoimmune illnesses, including psoriasis (85). Moreover, TQ exhibits a significant level of safety, even when administered at a dosage of 90 mg/kg (111). Nevertheless, due to its limited solubility in water and sensitivity to light, using it topically using traditional formulae does not yield any advantages (112). In order to address this issue, the development of a novel transdermal medication delivery method is necessary. Nanoparticles have been observed to increase the solubility and permeability of materials (111). Therefore, an experiment was conducted to investigate the efficacy of thymoquinone-loaded ethosomal vesicles in hydrogels utilizing the tail model for psoriasis (112). A study by Dwarampudi et al. (2012) showed that the ethanolic extract of *N. sativa* seeds showed significant epidermal differentiation compared to the negative control. This was equivalent to the effect of tazarotene gel. The 95% ethanolic extract showed good antiproliferative activity compared to Asiaticoside as the positive control (3). Their study demonstrated encouraging outcomes regarding the anti-psoriasis properties of TQ. Khaleghi et al. assessed the impact of black seed essential oil dietary supplementation on skin wound healing in goldfish, showing improved outcomes with a 6% concentration compared to a lower concentration (113). However, the TQ compound helps reduce inflammation and soothe irritated skin, making it an ideal ingredient for individuals with conditions like acne, eczema, or rosacea (114).

## 5 Recent research on nano-thymoquinone formulations in psoriasis

Nano-TQ formulations have shown promise in treating psoriasis, a chronic inflammatory skin disorder marked by excessive growth of keratinocytes and inflammation (112, 115). Several studies are focused on advancing nanomedicine methodologies to enhance the delivery and efficacy of TQ in psoriasis treatment (10, 116).

A recent study explored the effectiveness of nano-TQ-loaded lipid vesicles as a topical therapy for psoriasis. Lipid vesicles facilitate the specific delivery of TQ, a benzoquinone that readily dissolves in lipids, to the specific affected areas of the skin. Another innovative approach involves using NLCs co-encapsulating tacrolimus and TQ, termed TAC-THQ-NLCs. This nano gel aims to enhance the active compounds’ percutaneous delivery and anti-psoriatic efficacy of the active compounds of *N. sativa* seeds to refine topical psoriasis treatment. The synergistic combination of tacrolimus, an immunosuppressive agent, and TQ’s anti-inflammatory properties presents a potentially superior therapeutic strategy for managing psoriasis symptoms. Furthermore, the TAC-THQ-NG demonstrated significantly higher dose-dependent toxicity against a HaCaT cell line in comparison to a TAC-THQ suspension gel (TAC-THQ-SG). Compared to the suspension gel, the dye-loaded nanogel had better skin penetration depth, according to confocal microscopy (10).

Due to its limited bioavailability, TQ has only been the subject of two phase 1 clinical investigations. Nanotechnology is being explored to address this issue by creating TQ nanoparticles with higher bioavailability than free TQ. The success of nanocarrier compositions in enhancing the bioavailability of substances like curcumin and paclitaxel provides a precedent, and nanotechnology may offer a similar solution for TQ. This approach has been successful in the testing and eventual Food and Drug Administration (FDA) clearance of substances with poor bioavailability (80). The efficiency of *N. sativa* products for treating multiple skin disorders was evaluated using a meta-analysis done by Nasiri et al. (117), which included 14 randomized controlled trials. The study participants exhibited various forms of dermatological conditions, including psoriasis, eczema, and acne. Products derived from *N. sativa* significantly alleviated skin disease symptoms, according to a meta-analysis. **Table 2** is referenced, presumably containing information about various nano-TQ formulations in treating psoriasis.

**Table 2:** Types of nano-thymoquinone formulations in psoriasis treatment.

Type of nano-formulation	Biological vs. synthetic	Dose	Effects	Ref.
TQ-loaded dermal lipid nanoparticles	Synthetic	5 mg	• The main irritation index score and PASI score showed that psoriatic model had less erythema, edema, and thickness than the control group.	(118)
Tacrolimus and TQ co-loaded nanostructured lipid carriers	Synthetic	–	• The optimized TAC-THQ-NLC-based nanogel showed good physical properties, sustained drug release, higher toxicity against HaCaT cell line, and improved permeation depth in the skin.	(10)
TQ-Loaded Topical Nanoemulgel	Biological	0.5% (w/w)	• Improved skin penetrability and deposition characteristics, leading to quicker and early wound healing in preclinical study, comparable to a marketed silver sulfadiazine cream.	(109)
TQ-loaded ethosomal vesicles (benzoquinone)	Biological	Phospholipon 90G (3-4%) and TQ (200 mg) dissolved in ethanol (10-30%)	• The optimized composition of TQ-loaded ethosomal vesicles (EVs) demonstrated excellent pliability and possessed a spherical morphology within the nanoscale range. • TQ-loaded EVs exhibited significant retention in the layers of the skin and showed superior anti-psoriatic efficacy in comparison to plain thymoquinone.	(112)
Lipospheres of TQ	Synthetic	–	• Nitric oxide and pro-inflammatory cytokines IL-2, IL-6, IL-1β, and TNF-α were shown to be reduced in *in vitro* cell line tests. The phenotypic and histological characteristics of psoriatic skin improved in the *in vivo* study, and the levels of IL-17 and TNF-α were lowered. • Reduction in the intensity of symptoms was observed on the 6th day of treatment.	(105)

In ongoing research efforts, scientists are exploring the development of a nanoemulgel that combines TQ with fulvic acid extracted from peat. This combined therapy aims to leverage the anti-psoriatic effects of both components, potentially offering a novel and effective topical treatment for individuals with psoriasis. The nanoemulgel formulation is designed to enhance the permeation of TQ through the skin, amplifying its therapeutic effects (116). Vesicular systems based on phospholipids with an ethanol bilayer allow for increased drug loading of hydrophobic drugs and deeper penetration into the skin. Combining TQ with ethosomal vesicles facilitates deeper skin penetration, prolonged release, and an elevation in the therapeutic concentration of the medication at the site of injury (112). Furthermore, researchers are currently studying a nanoemulsion gel that has the ability to target two specific areas and contains both TQ and fulvic acid. This gel is being examined for its potential to improve the treatment of psoriasis and obtain better therapeutic results. This targeted delivery aims to maximize therapeutic effects by delivering both drugs to the affected psoriatic skin layers, potentially offering a more effective treatment strategy (116). Animal studies using BALB/c mice have shown promising results with nano-formulated TQ, demonstrating its efficacy in treating psoriasis while minimizing side effects (115).

The TQ-loaded lipid vesicle system is highlighted as an advanced formulation to improve the transportation of TQ directly to the affected areas of the skin, providing a more targeted and efficient approach to therapy for individuals with psoriasis (36). In summary, developing nano-TQ formulations holds significant potential in enhancing psoriasis treatment. These nanoformulations, including NLCs, lipid vesicles, and nanoemulgels, are designed to improve TQ’s administration, effectiveness, and safety as a topical treatment for psoriasis. Researchers are leveraging nanotechnology to develop more efficient and precise therapies with the potential to significantly enhance the quality of life for patients dealing with this persistent skin condition (10, 116).

## 6 Future directions

The challenges associated with the poor bioavailability of TQ have led to increased interest in leveraging nanotechnology to enhance its therapeutic application. Drugs like curcumin and paclitaxel have undergone similar approaches to improve bioavailability (80). In the case of TQ, additional preclinical investigations are needed to assess the pharmacokinetics, biodistribution, and toxicity profiles of various nano-TQ formulations. Dose-response studies should be conducted to determine the most effective doses for treating psoriasis while minimizing potential harm. Experimental studies using nano-TQ on different animal models, such as the imiquimod-induced psoriasis mouse model, could provide more evidence of its effectiveness. Robust clinical trials are essential to assess the safety, tolerability, and efficacy of nano-TQ formulations in individuals with varying degrees of psoriasis severity. Topical and oral nano-TQ should be evaluated as monotherapy or combined with other treatments. Longitudinal studies are crucial for understanding treatments’ long-term effectiveness and safety, especially in chronic conditions like psoriasis. By evaluating the durability of response over an extended period, researchers can assess whether alternative therapies, such as steroids, vitamin D analogs, and calcineurin inhibitors, provide sustained benefits and how they compare to traditional topical treatments.

Additionally, investigating the use of nano-TQ as a supplement to systemic and biologic treatments is an innovative approach. This may offer additional advantages, potentially enhancing the overall efficacy of treatment regimens for psoriasis. The utilization of nanotechnology to address the bioavailability challenges of TQ represents a promising avenue for improving the therapeutic outcomes in psoriasis patients.

Developing nano-TQ holds promise as a novel therapeutic approach for psoriasis, but substantial further research is necessary to harness its potential fully. Future investigations should prioritize optimizing nanocarrier design by:

1. Enhancing skin penetration: This allows more profound delivery of TQ to the affected area.
2. Facilitating controlled release: Sustained release ensures constant drug presence and improves treatment efficacy.
3. Targeting specific pathogenic immune cells: This focuses therapeutic action on the key drivers of inflammation in psoriasis, such as Th17 cells, Th1 cells, and Tc17 cells.

Novel delivery platforms like SLNs, microemulsions, liposomes, and NLCs deserve exploration for their potential to address these challenges. Combination therapy, where TQ is co-delivered with other anti-psoriatic drugs within a single nanoformulation, also presents significant opportunities. However, further substantial study is needed to fully explore and exploit its potential benefits. Future research endeavors should evaluate the pharmacokinetics, biodistribution, toxicity profiles, optimal dosing, and efficacy in diverse animal models. Rigorous and well-designed clinical trials are imperative to establish nano-TQ formulations’ safety, tolerability, and effectiveness. These trials should encompass patients with varying psoriasis severity, exploring mono and combination therapy approaches and, additionally, exploring novel nanocarrier designs.

## 7 Conclusion

Psoriasis is a chronic immune-mediated inflammatory skin disease affecting approximately 2-3% of the global population. While various treatment options exist, limitations include side effects, cost, diminished efficacy over time, and patient non-responsiveness. TQ, derived from *N. sativa* seeds, exhibits promising anti-inflammatory, antioxidant, and immunomodulatory properties that may benefit treating psoriasis. However, its limited water solubility and low absorption in the body have impeded its progression as a therapeutic agent. Recent studies have focused on nano-TQ formulations, encompassing nanoemulsions, lipid vesicles, NLCs, and ethosomes, to enhance the delivery and effectiveness of TQ in psoriasis treatment. Nanocarriers contribute to improved solubility, skin permeation, prolonged release, and targeted localization of TQ. Preclinical research utilizing mouse models has demonstrated the efficacy of nano-TQ in reducing inflammation, erythema, scaling, epidermal thickness, and cytokine levels such as TNF-α and IL-17 in psoriatic lesions. Synergistic benefits have been observed with the coadministration of TQ and tacrolimus. While clinical studies are currently limited, the promising results from *in vitro* and animal models warrant further investigation into the topical use of nano-TQ formulations as a novel approach for psoriasis treatment.
